# Generalized Pustular Psoriasis of Pregnancy Successfully Treated With Certolizumab Pegol: A Case Report and Literature Review

**DOI:** 10.7759/cureus.59832

**Published:** 2024-05-07

**Authors:** Dimitrii Pogorelov, Anne Tschesche, Galina Balakirski, Silke C Hofmann

**Affiliations:** 1 Center for Dermatology, Allergology and Dermatosurgery, Helios University Hospital Wuppertal, Wuppertal, DEU

**Keywords:** biologics, tnf-alpha inhibitor, certolizumab pegol, impetigo herpetiformis, generalized pustular psoriasis of pregnancy

## Abstract

Generalized pustular psoriasis of pregnancy (GPPP) is a rare dermatological condition that significantly affects maternal health and pregnancy outcomes. The treatment of this disease might be very challenging, as only a limited number of effective therapeutic options are available. If the use of systemic drugs is considered, they should ideally effectively control the systemic inflammation without harming the fetus. Here, we report the successful treatment of a severe case of GPPP in a 28-year-old woman using the tumor necrosis factor-alpha inhibitor (TNFi) certolizumab pegol. Additionally, we review the existing literature on the use of this class of drugs for treating GPPP. To date, there are only 11 reported cases of this severe skin condition treated with a TNFi. We also discuss the pathogenesis of GPPP and the rationale behind using TNFi for its treatment.

## Introduction

Generalized pustular psoriasis of pregnancy (GPPP), also known as impetigo herpetiformis, has traditionally been referred to as a distinct dermatological condition occurring predominantly in the third trimester of pregnancy or the postpartum period [1]. However, it may manifest as a variant of generalized pustular psoriasis (GPP) or evolve from preexisting plaque psoriasis during pregnancy [2]. GPPP presents significant risks to both the mother and the fetus, associated with increased chances of placental insufficiency, intrauterine growth retardation, congenital anomalies, and even stillbirth [3]. A mutation in the *IL36RN* gene, which encodes the interleukin-36 (IL-36) receptor antagonist protein, has been implicated in the pathogenesis of GPPP [4]. This condition is characterized by the emergence of sterile, centrifugal, coalescing pustules on an erythematous base, typically affecting skin folds. Accompanying systemic symptoms may include fever, neutrophilia, electrolyte imbalances, and elevated serum inflammatory markers. Although systemic corticosteroids (SCSs) are frequently used as a first-line treatment due to their favorable safety profile for fetuses [5], their efficacy in treating GPPP remains limited. There are also literature reports on the successful use of ciclosporin A (CsA), methotrexate, phototherapy, and granulocyte and monocyte adsorption apheresis in GPPP cases resistant to steroids [5,6]. In cases where these treatments fail and the disease progresses severely, biologics, including secukinumab [7] and tumor necrosis factor-alpha inhibitors (TNFis), have been found to rapidly control GPPP flares. Here, we report a severe case of GPPP that resolved completely following the initiation of certolizumab pegol (CTZ) and review the literature on the efficacy of CTZ and other TNFis in treating this disorder.

## Case presentation

**Figure 1:**
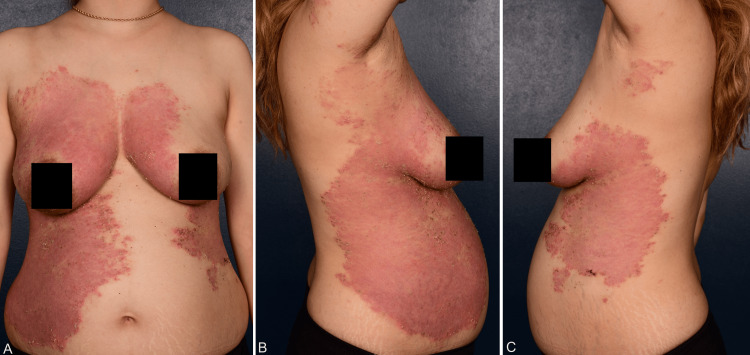
Clinical presentation of generalized pustular psoriasis of pregnancy occurring over the breast, abdomen (A), and the sides of the trunk (B, C) with typical erythematous plaques with peripheral crusts, circinate scaly rims, and widespread sterile pustules in the 24th week of gestation.

**Figure 2:**
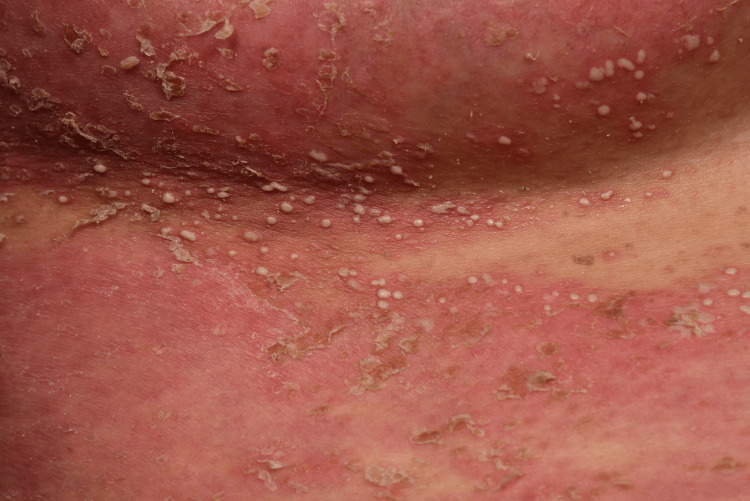
Detailed presentation of submammary skin lesions with multiple pustules on erythematous skin.

A 28-year-old woman of Middle Eastern descent presented in the 12th week of pregnancy (WP) with disseminated confluent pustules of variable size on an erythematous base with peripheral crusts and desquamation. The body surface area was about 19% (Figures 1A-1C, Figure 2). Her obstetric history was gravida 3, para 2. The patient reported no history of plaque psoriasis or GPP but recalled a pustular rash in her previous two pregnancies that was easily controlled by topical corticosteroids (TCSs).

**Figure 3:**
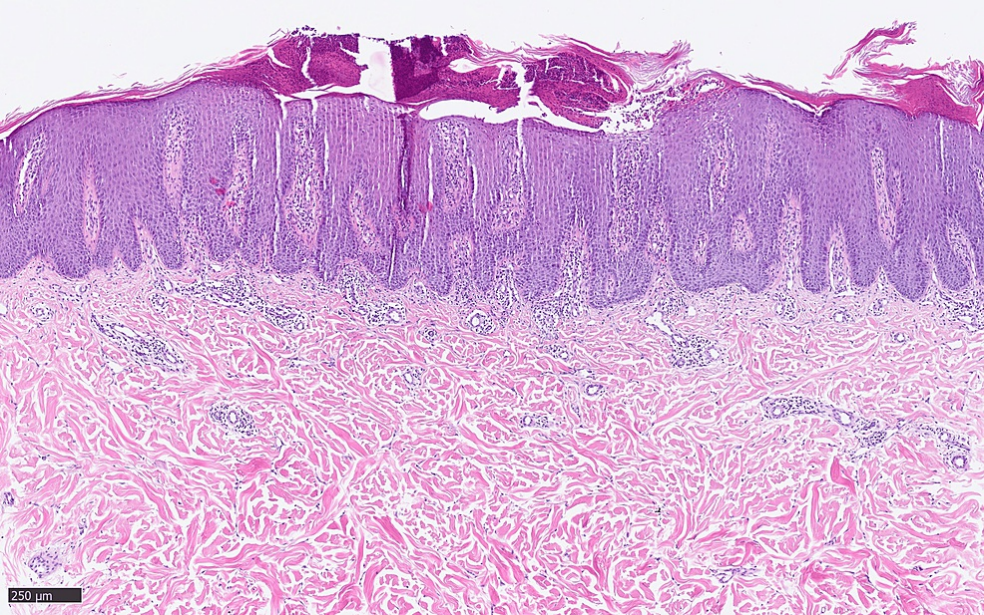
Histological finding (hematoxylin and eosin staining) from the lesional biopsy showing the characteristic findings of psoriasis with regular acanthosis of the rete ridges and parakeratosis (100×).

**Figure 4:**
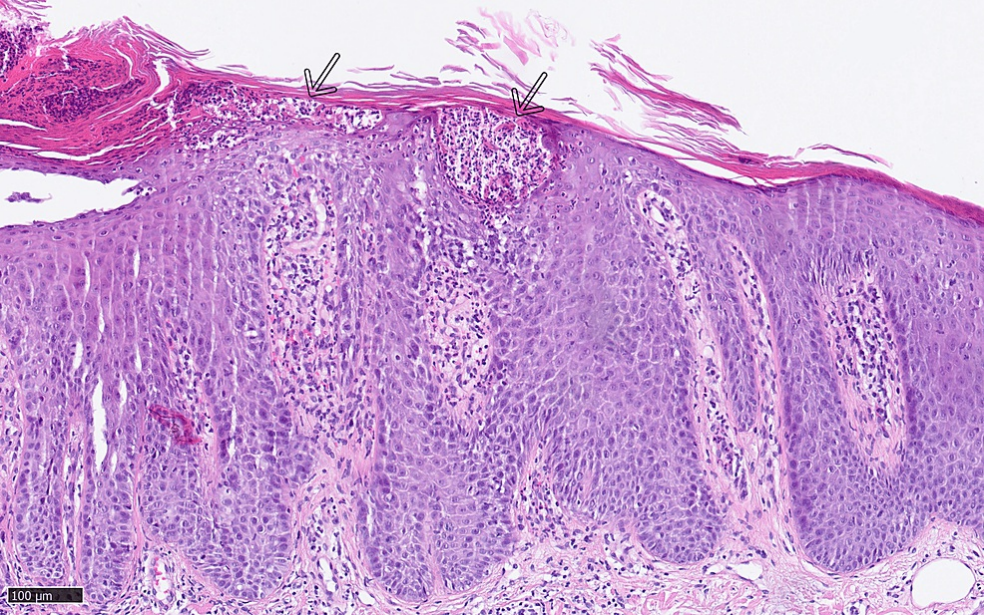
Histological finding (hematoxylin and eosin staining) from the lesional biopsy showing the characteristic subcorneal vesicle with neutrophils (pustule) labeled with arrows (200×).

Histologically, an acanthotic enlarged squamous epithelium and sub- and intracorneal pustules typical of GPP were noted (Figures 3, 4).

Standard laboratory workup showed mild leukocytosis, neutrophilia, and elevated C-reactive protein. Serum calcium was within the normal range, but serum concentration of 25-OH vitamin D was low (Table 1).

**Table 1:** Summary of the laboratory parameters of the patient, which differ from the norm.

Laboratory parameter	Laboratory results	Normal range	Units
Leukocytes	12.41	4.5–10.0	Cells/nL
Neutrophils, absolute count	10.12	1.9–8.0	Cells/nL
Neutrophils, relative count	81.6	45.0–75.0	%
C-reactive protein	7.8	<0.5	mg/dL
25-OH vitamin D	15.6	≥30.0	ng/mL

**Figure 5:**
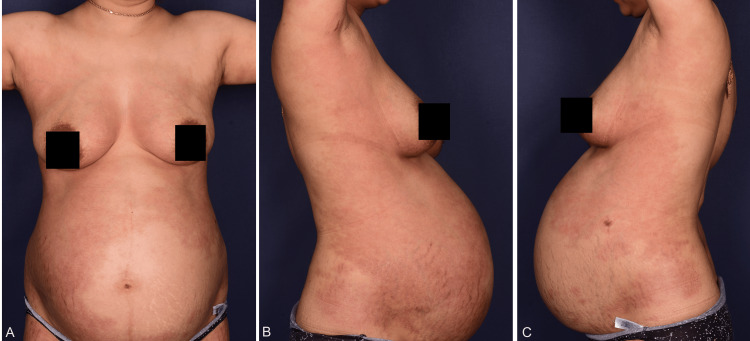
Resolution of skin lesions four weeks after the initiation of certolizumab pegol with post-inflammatory hyperpigmentation (A-C).

Initially, the patient was started on TCSs which led to only temporary symptom relief. We refrained from administering SCSs due to poorly controlled gestational diabetes. The severity of her skin condition, pruritus, and pain prompted us to initiate systemic therapy with CTZ at 30th WP. CTZ therapy was administered subcutaneously at 400 mg at weeks zero, two, and four, followed by 200 mg every two weeks, which is the standard dose approved for the treatment of plaque psoriasis by both the U.S. Food and Drug Administration and the European Medicines Agency. This therapy led to a significant improvement four weeks after the beginning of the treatment (Figures 5A-5C). At 37+0 WP, preterm premature rupture of the membranes occurred and contractions started. She underwent a cesarean section and delivered a 2,680 g healthy male infant (Apgar score: 9/10/10).

Due to the complete remission of GPPP during treatment with CTZ, the TNFi was discontinued three months after the initiation of therapy (approximately five weeks after delivery). However, about two months after discontinuation of CTZ, the patient suffered a severe relapse of GPPP. CTZ was, therefore, reintroduced at the above-mentioned regimen, with excellent results. Currently, about five months after delivery, the patient is still on CTZ, breastfeeding the baby, and free of symptoms. We plan to continue the current therapy for at least a further three to six months.

## Discussion

While the pathophysiological role of TNF-α in plaque psoriasis is well established, IL-36 is believed to play a crucial role in generalized pustular psoriasis [8]. By binding to its receptor (IL-1RL2), IL-36 activates the nuclear factor-κB signaling pathway, leading to increased production of proinflammatory cytokines such as IL-1, IL-6, and IL-8 [4,8]. The IL-36 receptor antagonist (IL36RN) is a protein that counteracts the inflammatory effects of IL-36. Mutations in the *IL36RN* gene, which result in the impaired function of IL36RN, have been identified in numerous patients with GPP [4]. Currently, the IL-36 receptor antagonist spesolimab is the only biologic drug approved for treating GPP in the United States and Europe. However, it is not licensed for use during pregnancy [8] and is currently unavailable in Germany.

Although the precise mechanism of action of TNFi in GPP is not completely understood, these agents have demonstrated efficacy in treating GPP, and particularly GPPP. Our comprehensive review of the literature identified a total of 10 publications documenting the use of TNFi in 11 patients with GPPP (Table 2) [9-18]. Five of these reports originated from Japan. The majority of these patients (8/11) had a prior history of plaque psoriasis and/or GPP. In six of the 11 cases, infliximab (IFX) was administered as the sole biologic treatment, while adalimumab (ADA) was used in two cases. However, ADA and IFX were ineffective in treating GPPP and were replaced with CTZ in the cases reported by Fukushima et al. [9] and Mizutani et al. [10], respectively. Similarly, the patient described by Post et al. [11] did not respond to either IFX or ADA but achieved recovery with CTZ.

**Table 2:** Summary of the literature on the use of TNF-α inhibitors in generalized pustular psoriasis of pregnancy. TNFi = tumor necrosis factor-alpha inhibitor; TNF-α = tumor necrosis factor-alpha; GPP = generalized pustular psoriasis; TCS = topical corticosteroids; SCS = systemic corticosteroids; CsA = ciclosporin A; ADA = adalimumab; IFX = infliximab; IVIG = intravenous immunoglobulin; SEC = secukinumab; CTZ = certolizumab pegol; GMA = granulocyte-monocyte apheresis; FAE = fumaric acid esters; ACT = acitretin; WP = weeks of pregnancy; ECS = elective cesarean section; EmCS = emergency cesarean section; SVD = spontaneous vaginal delivery; FTD = full-term delivery

Authors, year, reference	Country	Age (years)	Obstetric history	Onset/Exacerbation (weeks of pregnancy)	Preexisting plaque psoriasis (Pso) or GPP	Previous treatments (including therapy before pregnancy)	TNFi	Response to TNFi	Pregnancy and birth outcome
Our case	Germany	28	G3, P2	12	No	TCS	CTZ	Resolution within 4 weeks	ECS at 37 WP, no inborn abnormalities, Apgar score of 9
Fukushima et al., 2021 [9]	Japan	26	G1, P0	30	Pso, GPP	SCS, CsA, GMA, ADA	CTZ	Improvement within a few days	ECS at 37 WP, low birth weight, no inborn abnormalities, Apgar score of 8
Fukushima et al., 2021 [9]	Japan	35	G2, P1	20	No	SCS, CsA, GMA	ADA	Improvement	ECS at 37 WP, low birth weight, no inborn abnormalities, Apgar score of 8
Mizutani et al., 2020 [10]	Japan	31	G2, P1	22	GPP	SCS, CsA, GMA, IFX	CTZ	Subsequent resolution	SVD at 36 WP, low birth weight, no inborn abnormalities, Apgar score of 8
Post et al., 2021 [11]	Germany	39	G1, P0	33	GPP	FAE, ACT, apremilast, IFX, ADA, SEC	CTZ	Resolution	FTD, no inborn abnormalities, normal postnatal development
Beksac et al., 2021 [12]	Turkey	22	G2, P1	20	Pso	SCS, antibiotics	IFX	Resolution within 2 days	ECS at 36 WP, low birth weight, no inborn abnormalities, Apgar score of 10
Kobaner and Ekinci, 2020 [13]	Turkey	24	G1, P0	27	Pso, GPP	SCS, CsA, antibiotics	IFX	Resolution within 6 weeks	ECS at 40 WP, no inborn abnormalities
Ogrum et al., 2019 [14]	Turkey	25	G1, P0	18	GPP	SCS, CsA, IVIG, ACT	IFX	Improvement	ECT at 36 WP due to oligohydramnios, no inborn abnormalities
Adachi et al., 2016 [15]	Japan	18	G1, P0	8	Pso, GPP	GMA	IFX	Improvement after the first injection	EmCS at 38 WP due to early membrane rupture, fetal infection, normal postnatal development
Yamashita et al., 2019 [16]	Japan	32	G3, P2	18	No	SCS, GMA, dapsone	ADA	Resolution after 6 injections	FTD, no inborn abnormalities
Sheth et al., 2009 [17]	UK	27	G2, P1	20	No	SCS, UVB, CsA	IFX	Resolution after 3 injections	SVD at 36 WP, labor induction, no inborn abnormalities
Puig et al., 2010 [18]	Spain	28	G2, P1	34	Pso	TCS, SCS, CsA	IFX	Resolution within 6 weeks	ECS at 34–35 WP, no inborn abnormalities, normal postnatal development

In our patient, unlike all other reported cases, no other systemic therapy was used before or alongside the administration of CTZ. Seven of the 11 previously described patients received CsA before or during pregnancy (Table 2). However, the advantage of biologics over CsA, an immunosuppressive drug with known efficacy for GPPP, results from its possible side effect of inducing severe hypertension in pregnancy [19]. Among all biologic drugs used to treat psoriasis, TNFis have the most robust evidence supporting their safety during pregnancy [19]. Notably, the prenatal histories were unremarkable in all cases reviewed, and the patients receiving TNFis delivered healthy babies.

CTZ, a PEGylated TNFi, is the only biologic approved for treating plaque psoriasis during pregnancy and breastfeeding. Unlike other drugs in this class, it does not actively cross the placenta or get secreted into breast milk [19,20]. This distinctive pharmacological characteristic can be explained by its molecular structure, which consists only of the antigen recognition region of the antibody. The transfer of maternal immunoglobulin G (IgG) antibodies to the fetus across the placenta is mediated by the neonatal Fc receptor (FcRn). IgG antibodies bind to the FcRn via their Fc-fragment to cross the placenta. Due to its Fc-free molecular structure, CTZ cannot undergo FcRn-dependent placental transfer, unlike other TNFis [20].

Our case alongside the reviewed literature (Table 2) suggests that CTZ offers a potentially well-tolerated treatment option for GPPP. The decision regarding the duration of treatment and its discontinuation post-pregnancy should be tailored to the individual based on disease severity and the presence of preexisting plaque psoriasis or GPP, which may necessitate long-term therapy beyond the pregnancy.

## Conclusions

The therapy of GPPP might be challenging, as conventional drugs such as SCS or CsA may cause adverse events or be contraindicated if pregnancy is complicated by gestational diabetes or hypertension. Although IL-36 receptor antagonist spesolimab is approved for treating GPP, it is not licensed for use in pregnant women. At the same time, no further biologic drugs are licensed and available for GPP. This situation poses a therapeutic dilemma. On the other hand, some patients with GPP show an adequate response to TNFi treatment. Furthermore, among the currently available biologics, the largest evidence for safety during pregnancy exists for TNFi. These substances show a favorable safety profile and good therapeutic effect on GPPP, of which we preferred CTZ due to its approval for pregnant and breastfeeding women.
